# Ambient Air Pollution and Pregnancy Outcomes: A Review of the Literature

**DOI:** 10.1289/ehp.6362

**Published:** 2005-01-04

**Authors:** Radim J. Šrám, Blanka Binková, Jan Dejmek, Martin Bobak

**Affiliations:** ^1^Laboratory of Genetic Ecotoxicology, Institute of Experimental Medicine, Academy of Sciences, and Health Institute of Central Bohemia, Prague, Czech Republic; ^2^Department of Epidemiology and Public Health, University College London, London, United Kingdom

**Keywords:** air pollution, intrauterine growth retardation, low birth weight, molecular epidemiology, PAHs, particulate matter, PM_10_, premature birth, reproductive effects, SO_2_

## Abstract

Over the last decade or so, a large number of studies have investigated the possible adverse effects of ambient air pollution on birth outcomes. We reviewed these studies, which were identified by a systematic search of the main scientific databases. Virtually all reviewed studies were population based, with information on exposure to air pollution derived from routine monitoring sources. Overall, there is evidence implicating air pollution in adverse effects on different birth outcomes, but the strength of the evidence differs between outcomes. The evidence is sufficient to infer a causal relationship between particulate air pollution and respiratory deaths in the postneonatal period. For air pollution and birth weight the evidence suggests causality, but further studies are needed to confirm an effect and its size and to clarify the most vulnerable period of pregnancy and the role of different pollutants. For preterm births and intrauterine growth retardation (IUGR) the evidence as yet is insufficient to infer causality, but the available evidence justifies further studies. Molecular epidemiologic studies suggest possible biologic mechanisms for the effect on birth weight, premature birth, and IUGR and support the view that the relation between pollution and these birth outcomes is genuine. For birth defects, the evidence base so far is insufficient to draw conclusions. In terms of exposure to specific pollutants, particulates seem the most important for infant deaths, and the effect on IUGR seems linked to polycyclic aromatic hydrocarbons, but the existing evidence does not allow precise identification of the different pollutants or the timing of exposure that can result in adverse pregnancy outcomes.

There is extensive evidence that ambient air pollution affects human health (e.g., Brunekreef and Holgate 2002; Künzli et al. 2000; Pope et al. 2002). Most studies have focused on the effects of air pollution on adult mortality and respiratory morbidity (Dockery et al. 1993; Schwartz and Marcus 1990). However, some age groups appear to be more susceptible than others. For example, it has been shown that the effects are larger in the elderly than in the general adult population (Saldiva et al. 1995). Studies on childhood health risks, such as respiratory symptoms or hospital admissions for asthma, suggest that the opposite end of the age spectrum is also more vulnerable to air pollution than is the general population (Dockery and Pope 1994; Heinrich et al. 1999; Schwartz et al. 1994). In addition to these “traditional” end points in children, there is now emerging evidence that air pollution is also associated with elevated risk of adverse pregnancy outcomes (Glinianaia et al. 2004; Maisonet et al. 2004).

The study of birth outcomes is an important emerging field of environmental epidemiology. Birth outcomes are important in their own right because they are important indicators of the health of the newborns and infants. In addition, low birth weight (LBW), intrauterine growth retardation (IUGR), and impaired growth in the first years of life are known to influence the subsequent health status of individuals, including increased mortality and morbidity in childhood and an elevated risk of hypertension, coronary heart disease, and non-insulin-dependent diabetes in adulthood (Barker 1995; Osmond and Baker 2000).

It is increasingly apparent that there is a critical period of development when the timing of exposure and the dose absorption rate can be even more important for the biologic effects than is the overall dose (Axelrod et al. 2001). Fetuses, in particular, are considered to be highly susceptible to a variety of toxicants because of their exposure pattern and physiologic immaturity (Perera et al. 1999; Šrám 1999). Their developing organ systems can be more vulnerable to environmental toxicants during critical windows (sensitive periods of development) because of higher rates of cell proliferation or changing metabolic capabilities (Calabrese 1986). Therefore, prenatal exposure to environmental pollution can result in some adverse reproductive outcomes, similar to the association between maternal active and passive smoking and impaired reproductive outcomes (Misra and Nguyen 1999; Salihu et al. 2004). The specific mechanisms that may account for the link between ambient air pollution and adverse reproductive outcomes are also reviewed in this article.

The objective of this review is to examine the evidence linking adverse birth outcomes with ambient air pollution. For the purpose of this review, birth outcomes have been divided into five groups: *a*) mortality of fetuses and infants, *b*) LBW, *c*) premature (preterm) births, *d*) IUGR, and *e*) birth defects. In this article we review the evidence on each of these separately. For each of the outcomes, we assess the three critical issues in interpreting epidemiologic studies (random error, selection or measurement bias, and confounding); issues related to all reviewed outcomes (e.g., publication bias or biologic plausibility) are considered together at the end of the article. By weighting the evidence, we attempt to draw balanced conclusions about the relations between air pollution and birth outcomes.

## Materials and Methods

We searched all publications included in the electronic databases PubMed (from 1966; National Library of Medicine, Bethesda, MD, USA) and the Science Citation Index and Social Science Citation Index of the Institute of Scientific Information, available on the Web of Knowledge (from 1981; Thompson Scientific, Philadelphia, PA, USA). We searched for combinations of either of the key words “air pollution” or “pollution” with any of the following: “infant mortality,” “postneonatal mortality,” “post-neonatal mortality,” “birth weight,” “birth-weight,” “intrauterine growth retardation,” “IUGR,” “premature birth,” “prematurity,” “fetal growth,” and “foetal growth.” We also searched the reference lists of identified papers for additional publications. We excluded abstracts of conference presentations because they did not contain sufficient information (but relevant conference abstracts that were subsequently published as full papers were included).

## Results

### Air pollution and childhood mortality.

The possible impact of air pollution on children’s health was first connected to early child mortality. One of the earliest reports was based on an ecologic study of counties of England and Wales in 1958–1964, with air pollution estimated from indices of domestic and industrial pollution (Collins et al. 1971). The study found significant correlations between air pollution and infant mortality, and infant respiratory mortality in particular. The Nashville Air Pollution Study, conducted in the 1950s, indicated that dust fall, a measure of air pollution estimated for each census tract, was related to neonatal deaths with signs of prematurity, but the results were inconclusive (Sprague and Hagstrom 1969). Another early signal that air pollution may be associated with deaths in infancy came from the extensive analyses of air pollution and mortality in 117 U.S. metropolitan areas in the 1960s (Lave and Seskin 1977). Particulates and, to a lesser degree, sulfate concentrations were positively associated with infant mortality; a 10% increase in pollution was associated with a 1% increase in infant mortality.

Almost two decades passed before a new generation of studies addressed this question in more detail. These newer studies, summarized in Table 1, confirmed the early results. A small ecologic study in the Rio de Janeiro, Brazil, metropolitan area reported a positive association between annual levels of particulates and infant mortality from pneumonia (Penna and Duchiade 1991).

Bobak and Leon (1992) studied infant mortality in an ecologic study in the Czech Republic. The study found an association between sulfur dioxide and total suspended particles (TSP), and infant mortality, after controlling for a number of potential confounding variables (at the ecologic level). The effects were specific to respiratory mortality in the postneonatal period. These results were later confirmed in a nationwide case–control study based on the Czech national death and birth registers; this design allowed controlling for social and biologic covariates at the individual level (Bobak and Leon 1999a). The study found a strong effect of SO_2_ and TSP on postneonatal mortality from respiratory causes: the relative risks (RRs) per 50 μg/m^3^ increase in pollutant concentration were 1.95 [95% confidence interval (CI), 1.09–3.50] for SO_2_ and 1.74 (95% CI, 1.01–2.98) for TSP.

Woodruff et al. (1997) analyzed the association between early postneonatal mortality and PM_10_ (particulate matter < 10 μm) levels in about 4 million babies born from 1989 through 1991 in the United States. Infants were categorized as having high, medium, or low exposures based on tertiles of PM_10_. After adjustment for other covariates, for total postneonatal mortality for the high-exposure (> 40 μg/m^3^) versus low-exposure (< 28 μg/m^3^) groups was 1.10 (95% CI, 1.04–1.16). In infants of normal birth weight, high PM_10_ exposure was associated with respiratory deaths (RR, 1.40; 95% CI, 1.05–1.85) and sudden infant death syndrome (RR, 1.26; 95% CI, 1.14–1.39). The results were similar in term and LBW infants.

Pereira et al. (1998) investigated the associations between daily counts of intrauterine mortality in the city of São Paulo, Brazil, in 1991–1992 and daily measurements of several pollutants: nitrogen dioxide, SO_2_, carbon monoxide, ozone, and PM_10_. The association was strongest for NO_2_ (coefficient *R* = 0.0013/μg/m^3^; *p* < 0.01). A significant association was also observed with exposure combining the pollutants NO_2_, SO_2_, and CO together (*p* < 0.01).

Loomis et al. (1999) conducted a time-series study of infant mortality in the southwestern part of Mexico City in 1993–1995. Exposure included NO_2_, SO_2_, O_3_, and particulate matter < 2.5μm (PM_2.5_). A 10 μg/m^3^ increase in the mean level of fine particles during the preceding 3 days was associated with a 6.9% (95% CI, 2.5–11.3%) excess increase in infant death.

Dolk et al. (2000) examined perinatal and infant outcomes in populations residing near 22 coke works in Great Britain. Data on specific pollutants were not provided; the exposure was based on the proximity to pollution sources. The ratios of observed to expected cases for residence in proximity of the coke works were 0.94 (95% CI, 0.78–1.12) for stillbirth, 0.95 (95% CI, 0.83–1.09) for infant mortality, 0.86 (95% CI, 0.72–1.03) for neonatal mortality, 1.10 (95% CI, 0.90–1.33) for postneonatal mortality, 0.79 (95% CI, 0.30–1.46) for respiratory postneonatal mortality, and 1.07 (95% CI, 0.77–1.43) for sudden infant death syndrome in the postneonatal period. This study, however, had a limited statistical power owing to the relatively small size of the study.

A time-series analysis of daily deaths in Seoul, South Korea, found a relatively specific association between PM_10_ and total and respiratory mortality in the postneonatal period; the RRs per 10 μg/m^3^ were 1.03 (1.02–1.04) and 1.18 (1.14–1.21), respectively (Ha et al. 2003).

The consistency of these studies, conducted in a range of different populations and using both spatial and time-series study designs, is remarkable. The three largest studies produced very similar estimates of RR (Bobak and Leon 1992, 1999a; Woodruff et al. 1997). Perhaps the only alternative explanation that may affect the interpretation of these studies is confounding by maternal smoking. It is likely that maternal smoking is associated with children’s risk of respiratory death, and none of the studies was able to control for maternal smoking on the individual level. However, at least three observations argue against this possibility. First, all recent studies controlled for socioeconomic factors and other potential confounders. Because smoking in industrialized countries is strongly socially patterned, adjustment for socioeconomic factors should at least partly adjust for smoking. This would be reflected by adjusted estimates being substantially smaller than unadjusted ones. However, in most instances the differences between the crude and adjusted effect estimates were minimal. This does not suggest a presence of residual confounding.

Second, the results of spatial and time-series studies were similar. It is highly unlikely that the social composition or maternal smoking in the studied populations would change substantially over the relatively short periods covered by the time-series studies. In our view, the time-series design practically precludes a presence of confounding by socioeconomic factors or maternal smoking.

Finally, the studies were conducted in very different populations, ranging from China to the United States and from Brazil to the Czech Republic; it is unlikely that the distribution of socioeconomic disadvantage or maternal smoking with respect to air pollution would be similar enough in these different countries to produce the same pattern of results. We therefore conclude that the evidence is sufficient to infer causal relationship between particulate air pollution and respiratory deaths in the postneonatal period.

### Air pollution and birth weight.

The potential effects of air pollutants on birth weight were first examined in a small case–control study by Alderman et al. (1987); the study did not find any relationship between neighborhood ambient levels of CO during the third trimester of pregnancy and LBW. Over the last decade, this question has been investigated in a number of studies (summarized in Table 2).

Wang et al. (1997) examined the effects of SO_2_ and TSP on birth weight in a time-series study in four relatively highly polluted residential areas of Beijing, China. A spectrum of potential confounding factors was adjusted for in multivariate analysis. A graded dose–effect relationship was found between maternal exposure to SO_2_ and TSP during the third trimester and infant birth weight. The mean birth weight was reduced by 7.3 and 6.9 g for each 100-μg/m^3^ increase in SO_2_ and TSP, respectively. The RRs of LBW associated with a 100-μg/m^3^ increase in SO_2_ and TSP were 1.11 (95% CI, 1.06–1.16) and 1.10 (95% CI, 1.05–1.14), respectively. The authors speculated that SO_2_ and particles, or some complex mixtures associated with these pollutants, during late gestation contributed to the LBW risk in the studied population.

Bobak and Leon (1999b) conducted an ecologic study of LBW and levels of nitrous oxides (NO_x_), SO_2_, and TSP in 45 districts of the Czech Republic in 1986–1988. After controlling for socioeconomic factors, the RRs of LBW associated with an increase of 50 μg/m^3^ in the annual mean concentrations were 1.04 (95% CI, 0.96–1.12) for TSP, 1.10 (95% CI, 1.02–1.17) for SO_2_, and 1.07 (95% CI, 0.98–1.16) for NO_x_. When all pollutants were included in one model, only SO_2_ remained related to LBW [odds ratio (OR), 1.10; 95% CI, 1.01–1.20].

In a subsequent study, Bobak (2000) analyzed individual-level data on all single live births in the Czech Republic that occurred in 1991 in the 67 districts where at least one pollutant (NO_x_, SO_2_, or TSP) was monitored. The risk of LBW was analyzed separately for each trimester of pregnancy. The association between LBW and pollution was strongest for pollutant levels during the first trimester of pregnancy. The RRs of LBW per 50 μg/m^3^ increase in the mean concentration of SO_2_ and TSP during the first trimester were 1.20 (95% CI, 1.11–1.30) and 1.15 (95% CI, 1.07–1.24), respectively.

In a population-based study in Southern California, Ritz and Yu (1999) examined the influence of pollution levels during the third trimester on LBW risk in a cohort of 126,000 term births. The exposure to O_3_, NO_2_, and PM_10_ in the last trimester was estimated from continuous monitoring data. After adjustment for potential confounders, the risk of LBW was associated with maternal exposure to > 5.5 ppm CO during the third trimester (RR, 1.22; 95% CI, 1.03–1.44). The association between LBW risk and pollution exposure during earlier gestational stages was not significant.

In a population-based case–control study in Georgia (USA), Rogers et al. (2000) analyzed the combined effect on very low birth weight (VLBW) (< 1,500 g) of SO_2_ and TSP levels, using annual exposure estimates. The risk of VLBW was increased in babies of mothers who were exposed to concentrations of the combined pollutants above the 95th percentile of the exposure distribution (56.8 μg/m^3^); the RR was 2.88 (95% CI, 1.16–7.13).

Maisonet et al. (2001) examined the association between term LBW and ambient levels of SO_2_, PM_10_, and CO in six large cities in the northeastern United States. Their results suggested that the effects of ambient CO and SO_2_ on the risk of term LBW may differ by ethnic group. In Caucasians (*n* ~ 36,000), the risk of LBW associated with a 10-ppm increase in SO_2_ was 1.18 (95% CI, 1.12–1.23) in the first trimester, 1.18 (95% CI, 1.02–1.35) in the second, and 1.20 (95% CI, 1.06–1.36) in the third. By contrast, in African Americans (*n* ~ 47,000) LBW was associated with CO; a 1-ppm increase in CO concentration was associated with an RR of 1.43 (95% CI, 1.18–1.74) in the first trimester and 1.75 (95% CI, 1.50–2.04) in the third trimester. No effects were seen in Hispanics (*n* ~ 13,000), although this may have been due to a lower statistical power in this group.

Lin et al. (2001b) compared the rates of adverse pregnancy outcomes in an area polluted by the petrochemical industry and in a control area in Taiwan. The exposed and control areas differed substantially in the levels of air pollution; for example, the differences in the mean concentrations of PM_10_ was 26.7 μg/m^3^. The RR of term LBW, when the petrochemical municipality was compared with the control municipality, was 1.77 (95% CI, 1.00–3.12).

Ha et al. (2001) examined full-term births from 1996 through 1997 in Seoul, South Korea, to determine the association between LBW and exposure to CO, SO_2_, NO_2_, TSP, and O_3_ in the first and third trimesters. They found that ambient CO, SO_2_, NO_2_, and TSP concentrations during the first trimester of pregnancy were associated with LBW; the RRs were 1.08 (95% CI, 1.04–1.12) for CO, 1.06 (95% CI, 1.02–1.10) for SO_2_, 1.07 (95% CI, 1.03–1.11) for NO_2_, and 1.04 (95% CI, 1.00–1.08) for TSP.

Vassilev et al. (2001b) used U.S. Environmental Protection Agency (EPA) Cumulative Exposure Project data to investigate the association between outdoor airborne polycyclic organic matter (POM) and adverse reproductive outcomes in New Jersey for newborns born in 1991–1992. The RR of LBW in term babies, comparing the highest and the lowest exposure groups, was 1.31 (95% CI, 1.21–1.43).

Bobak et al. (2001) tested the hypothesis that air pollution is related to LBW on data from a British 1946 cohort. They found a strong association between birth weight and air pollution index based on coal consumption. After controlling for a number of potential confounding variables, babies born in the most polluted areas (annual mean concentration of smoke > 281 μg/m^3^) were on average 82 g (95% CI, 24–140) lighter than those born in the areas with the cleanest air (mean smoke concentration < 67 μg/m^3^).

Chen et al. (2002) examined the association between PM_10_, CO, and O_3_ and birth weight in northern Nevada (USA) from 1991 through 1999. The results suggested that a 10-μg/m^3^ increase in the mean PM_10_ concentrations during the third trimester of pregnancy was associated a reduction in birth weight of 11 g (95% CI, 2.3–19.8).

Wilhelm and Ritz (2003) studied the effect on LBW of residential proximity to heavy traffic in Los Angeles County, California (USA) in 1994–1996. The risk of term LBW increased by 19% for each 1 ppm increase in the mean annual concentration of background CO. In addition, an elevated risk was observed for women whose third trimester fell during the fall/winter months (RR, 1.39; 95% CI, 1.16–1.67); this is probably due to the more stagnant air conditions during the winter period. Overall, the study reported an approximately 10–20% increase in the risk of term LBW in infants born to women exposed to high levels of traffic-related air pollution.

A time-series study in Vancouver, Canada, found that LBW was associated with SO_2_ in the first month of pregnancy (OR per 5 ppb increase, 1.11; 95% CI, 1.01–1.22); NO_2_, CO, and O_3_ were not independently associated with LBW (Liu et al. 2003). Particles were not measured.

A time-series study in Sao Paulo, Brazil, found that birth weight was inversely related to CO in the first trimester; after controlling for potential confounders, a 1-ppm increase in the mean CO concentration in the first trimester was associated with a 23-g (95% CI, 5–41 g) reduction in birth weight (Gouveia et al. 2004).

The results of studies of outdoor exposures are complemented by studies of indoor and personal exposures (not included in Table 2). Boy et al. (2002) compared the association between birth weight and the type of fuel (open fires with wood smoke, chimney stove, and electricity/gas) used in kitchens by mothers in rural Guatemala during pregnancy. The use of open fire produced average levels of 24-hr PM_10_ of about 1,000 μg/m^3^. Babies of mothers using wood fuel and open fires were on average 63 g (95% CI, 0.4–126 g) lighter than those of women using electricity/gas. Perera et al. (2003) evaluated the effects of prenatal exposure to airborne carcinogenic polycyclic aromatic hydrocarbons (PAHs) monitored during pregnancy by personal air sampling in a sample of 263 nonsmoking African-American and Dominican women in New York. The mean total PAH exposure was 3.7 ng/m^3^ (range, 0.4–36.5 ng/m^3^). Among African Americans, high prenatal exposure to PAHs was associated with lower birth weight (*p* = 0.003) and smaller head circumference (*p* = 0.01). No such effects were observed among Dominican women.

Several methodologic issues should be considered in the interpretation of these studies. First, could chance (random error) play a role here? In several of the studies reviewed above, there is a potential problem of multiple comparisons. The more comparisons that are made, the higher the probability that some of them will be “statistically significant.” In some instances, a more stringent use of statistical testing would be useful. Especially in studies where exposures to different pollutants at different pregnancy periods were analyzed, some of the associations could be chance findings. In addition, exposures in different pregnancy periods and concentrations of different pollutants are mutually correlated, and efforts to separate their effects are not reliable.

Second, as with infant mortality, confounding by socioeconomic factors and maternal smoking could affect the results. Overall, however, this seems unlikely for the same reasons as those listed above in “Air pollution and childhood mortality.” In addition, some of the studies were able to control for social conditions and maternal smoking at the individual level, and the results were essentially identical.

In terms of the magnitude of the effect, the results were consistent in suggesting that the effects are relatively small. For comparison, it has been estimated that active smoking in pregnancy leads to a reduction in birth weight by approximately 150–200 g (Adriaanse et al. 1996), and exposure to environmental tobacco smoke in pregnancy results in birth weight reduction by approximately 20–30 g (Windham et al. 1999). There were also substantial inconsistencies in the results with respect to the importance of individual pollutants and the timing of critical exposure. The extent of the inconsistencies was such that the studies were not “combinable” into a formal meta-analysis to produce pooled effect estimates, although it is possible that the mix of pollutants differs between different settings and that this underlies the discrepancies in results.

The evidence suggests causality of the effect of air pollution on birth weight. However, given the potential problem with multiple comparisons and the heterogeneity of results, further studies are needed to confirm that the effect is indeed causal, to clarify the most vulnerable periods of pregnancy and the role of individual pollutants, and to examine whether the impaired reproductive outcomes have any long-term consequences on child health.

### Air pollution and premature births.

Perhaps the first study that suggested a possible association between air pollution and preterm births was the Nashville Air Pollution Study; the results suggested that dust fall (a measure of particulate pollution) was associated with neonatal deaths among premature births (Sprague and Hagstrom 1969). However, the study did not address the question of preterm births specifically, and there were concerns about confounding by socioeconomic variables. It was only in the 1990s when this issue was investigated in more detail (Table 3).

The first “modern” investigation of the possible influence of air pollution on premature birth was a time-series study in Beijing, China, conducted by Xu et al. (1995). The study found an inverse relationship between gestational age and the concentration of SO_2_ and TSP; the RRs of premature birth associated with a 100-μg/m^3^ increase in the mean SO_2_ and TSP concentrations during pregnancy, after controlling for potential confounders, were 1.21 (95% CI, 1.01–1.45) and 1.10 (95% CI, 1.01–1.20), respectively. Trimester-specific effects were not studied.

Bobak (2000) examined the relation between premature birth and ambient NO_x_, SO_2_, and TSP during each trimester. The association was strongest for SO_2_, weaker for TSP, and only marginal for NO_x_. For exposures during the first trimester, the RRs associated with a 50-ng/m^3^ increase in pollutant concentrations were 1.27 (95% CI, 1.16–1.39) and 1.18 (95% CI, 1.05–1.31) for SO_2_ and TSP, respectively. The effects of pollutants on premature births in the later two trimesters were weak.

The possible impact of CO, NO_2_, O_3_, and PM_10_ on premature birth was studied by Ritz et al. (2000) in Southern California. After adjustment for a number of biologic, social, and ethnic covariates, premature births were associated with CO and PM_10_ in the first gestational month and during late pregnancy. The RR associated with PM_10_ during the first gestational month was 1.16 (95% CI, 1.06–1.26); exposure in the last 6 weeks of gestation was associated with an RR of 1.20 (95% CI, 1.09–1.33). The association of premature birth with CO levels was not consistent throughout the study area.

In a study in a petrochemically polluted area in Taiwan, Lin et al. (2001a) found an RR of preterm birth in the polluted area, compared with the clean area, of 1.41 (95% CI, 1.08–1.82), after controlling for potential confounders.

The Vancouver time-series study found that the risk of preterm birth was associated with SO_2_ and CO during the last month of pregnancy; the ORs were 1.09 (1.01–1.19 per 5-ppb increase) and 1.08 (1.01–1.15 per 1-ppm increase), respectively (Liu et al. 2003).

The interpretation of the studies of preterm birth is complicated by similar issues as in the case of birth weight: the issue of multiple comparisons, and the inconsistency of the results in terms of the role of individual pollutants and the timing of exposure. In addition, there have been fewer studies of premature birth than of birth weight. We therefore conclude that the evidence, as yet, is insufficient to infer causality, but further studies are justified.

### Air pollution and IUGR.

IUGR is defined as birth weight below the 10th percentile of birth weight for gestational age and sex. IUGR is an interesting end point that may predict functional changes in adulthood, such as hypertension and coronary heart disease. The studies of the relationship between IUGR and air pollution are summarized in Table 4.

Dejmek et al. (1999) examined the impact of PM_10_ and PM_2.5_ on IUGR in a highly polluted area of northern Bohemia (Teplice District). The mean concentration of pollutants in each month of gestation for each mother were estimated from continuous monitoring data. A significantly increased risk of giving birth to a child with IUGR was established for mothers who were exposed to PM_10_ levels > 40 μg/m^3^ or PM_2.5_ > 27 μg/m^3^ during the first month of gestation. The adjusted odds ratio (AOR) associated with a 10-μg/m^3^ increase in PM_10_ was 1.25 (95% CI, 1.08–1.56); a similar, although weaker, association was seen for PM_2.5_. There was no association between IUGR and particulate levels in later gestational months or with SO_2_, NO_x_, or O_3_ (Dejmek et al. 1996).

The question of IUGR was addressed again in a reanalysis of an extended data set (Dejmek et al. 2000). Compared with exposure to the mean PM_10_ of < 40 μg/m^3^ during the first month of gestation, the AOR was 1.44 (95% CI, 1.03–2.02) for the medium-exposure group (PM_10_ 40 to < 50 μg/m^3^) and 2.14 (95% CI, 1.42–3.23) for PM_10_ of ≥ 50 μg/m^3^. Using a continuous exposure, the AOR of IUGR was 1.19 (CI, 1.06–1.33) per 10-μg/m^3^ increase of PM_10_ in the first gestational month.

In further analyses of this cohort, Dejmek et al. (2000) investigated the association between carcinogenic PAHs and IUGR in two Czech districts: Teplice and Prachatice. In the Teplice data, there was a highly significant increase of IUGR with exposures to carcinogenic PAHs (benz[*a*]anthracene, benzo[*b*]fluoranthene, benzo[*k*]fluoranthene, benzo[*g*,*h*,*i*]perylene, benzo[*a*]pyrene, chrysene, dibenz[*a*,*h*]anthracene, and indeno[1,2,3-*c*,*d*]pyrene) above 15 ng/m^3^. Again, the effect was specific for the first gestational month. The AORs were 1.59 (95% CI, 1.06–2.39) for medium levels of carcinogenic PAHs and 2.15 (95% CI, 1.27–3.63) for high exposure levels. Using a continuous measure of exposure, a 10 ng/m^3^ increase in carcinogenic PAH level was associated with an AOR of 1.22 (95% CI, 1.07–1.39). Although there was no effect of PM_10_ on IUGR found in Prachatice, the association between carcinogenic PAHs and IUGR was close to that found in Teplice. Again, the only consistent association between carcinogenic PAHs and IUGR was observed in the first gestational month: compared with the lowest category of exposure to carcinogenic PAHs, the AOR of IUGR was 1.63 (95% CI, 0.87–3.06) in the medium category and 2.39 (95% CI, 1.01–5.65) in the highest category.

The analysis of the Czech national birth register linked with air pollution data did not reveal any significant association between IUGR and ambient levels of NO_x_, SO_2_, and TSP (Bobak 2000). The discrepancy between the Czech studies is probably related to exposure measurement. PAHs appear to be the critical exposure for IUGR, but PAHs were not measured by the national monitoring system used for exposure estimation by Bobak (2000).

Vassilev et al. (2001a) examined the association of POM in outdoor air with “small for gestational age” (SGA) births (the definition of SGA is identical to that of IUGR). Information from birth certificates in New Jersey (USA) from 1991 through 1992 was combined with data on air toxicity derived from the U.S. EPA Cumulative Exposure Project, using the predicted POM concentrations from annual exposure estimates. The AOR for term SGA in the highest exposure tertile (0.61–2.83 μg/m^3^, which includes about 89% of the state’s births) was 1.22 (95% CI, 1.16–1.27), suggesting that residential exposure to airborne POM is associated with an increased prevalence of IUGR.

In the Vancouver study, using the time-series approach, SO_2_, NO_2_, and CO in the first month of pregnancy were associated with IUGR; the ORs were 1.07 (95% CI, 1.01–1.13) per 5-ppb increase, 1.05 (95% CI, 1.01–1.10) per 10-ppb increase, and 1.06 (95% CI, 1.01–1.10) per 1-ppm increase, respectively (Liu et al. 2003). Data on exposures to particles or PAHs were not available in that study.

As with studies of birth weight and preterm births, the reviewed studies of IUGR produced inconsistent results, and the interpretation is complicated by multiple comparisons (Bobak 2000; Liu et al. 2003) and mutual correlations of exposures. The results by Dejmek et al. (1999, 2000) and Liu et al. (2003) suggest that the first month was the most sensitive period for the effect of air pollutants, but further studies should clarify this issue. Data by Dejmek et al. (2000) and Vassilev et al. (2001a) imply a critical role of PAHs. It is possible that carcinogenic PAHs are responsible for the biologic activity of complex mixtures adsorbed to respirable air particles that can result in IUGR. With the increase in traffic, the significance of PAHs in Europe is growing, but their monitoring remains scarce. At present, the evidence is insufficient to infer causality, but further studies are required.

### Air pollution and birth defects.

At present, the evidence on the relation between outdoor air pollution and birth defects is limited to only one report. Ritz et al. (2002) evaluated the effect of CO, NO_2_, O_3_, and PM_10_ on the occurrence of birth defects in Southern California for the period 1987–1993. The average monthly exposure for each pollutant throughout pregnancy was calculated. Dose–response patterns were observed for CO exposure in the second month of gestation and ventricular septal defects (AOR for the highest vs. lowest quartile of exposure, 2.95; 95% CI, 1.44–6.05) and for exposure to O_3_ in the second month and aortic artery and valve defects (AOR, 2.68; 95% CI, 1.19–6.05).

Given the lack of studies on air pollution and birth defects, the evidence base available so far is insufficient to draw conclusions about causality. Further studies are required to support these results by Ritz et al. (2002).

## Discussion

The studies reviewed above indicate that ambient air pollution is inversely associated with a number of birth outcomes. This is a relatively new area of environmental epidemiology; most reports have emerged over the last 10 years. A critical assessment of the evidence is therefore timely. Issues pertinent to different studies were considered separately above. Here, we consider questions common to all reproductive outcomes: publication bias, measurement of exposure, and the biologic plausibility of the effects on birth weight, IUGR, and preterm births.

### Publication bias.

Negative studies are less likely to be published, and studies published in non-English journals are less likely to be included in reviews. We included all studies we were able to identify. We cannot exclude the possibility that some negative studies, especially in the earlier period, remain unpublished. However, given the recent interest in this topic, it is likely that most studies over the last decade have been published or at least presented at conferences.

### Measurement of exposure.

Most studies relied on routine monitoring of air pollution in large areas. Extrapolation from citywide or areawide measurements to individual exposures can be problematic. In this context, molecular epidemiologic studies are particularly valuable for the interpretation of the epidemiologic data. The molecular epidemiologic studies used biomarkers of exposure, mainly as the DNA adducts measured by ^32^P-postlabeling and PAH–DNA adducts assessed by enzyme-linked immunosorbent assays (Šrám and Binková 2000). Overall, these studies suggest that DNA adduct levels in maternal blood and placentas are higher in areas with higher pollution levels (Šrám et al. 1999; Whyatt et al. 1998), and significant district and seasonal differences in DNA adducts were found in subgroups with the *GSTM1* null genotype (Topinka et al. 1997a, 1997b). The increase in the levels of DNA adducts related to pollution is similar to, but smaller in magnitude than, the differences between smoking and non-smoking mothers. All this indicates that ambient air pollution levels do translate to higher individual exposures, even for unborn babies. This provides support for the validity of the epidemiologic studies reviewed above.

DNA adducts in placentas and the impact of PAHs on IUGR are consistent with findings of *in vitro* studies that exposure to extracts of urban air PM increased DNA adducts and embryotoxicity (Binková et al. 1999, 2003). These findings indicate that particle-bound carcinogenic PAH concentrations may be taken as an index of the biologically active components in samples of particulates in air.

### Biologic plausibility.

The molecular epidemiologic studies suggest biologic mechanisms for the effect of air pollution on birth outcomes. It has been shown that the levels of DNA adducts are positively related to risk of IUGR (Dejmek at al 2000; Šrám et al. 1999), birth weight, birth length, and head circumference (Perera et al. 1998, 1999), and hypoxanthine-guanine phosphoribosyltransferase (*HPRT*) locus mutation frequency in infants (Perera et al. 2002).

PAHs and/or their metabolites may bind to the aryl hydrocarbon receptor (AhR) and accumulate in the nucleus of cells, resulting in increased rates of mutagenesis. Because PAHs bind to the AhR, it may result in anti-estrogenic activity through increased metabolism and the depletion of endogenous estrogens (Carpenter et al. 2002), thus disrupting the endocrine system by altering steroid function. Bui et al. (1986) hypothesized that benzo[*a*]pyrene exposure may interfere with uterine growth during pregnancy because of its antiestrogenic effects, thereby disrupting the endocrine system. Fetal toxicity may be further caused by DNA damage resulting in activation of apoptotic pathways (Nicol et al. 1995) or binding to receptors for placental growth factors resulting in decreased exchange of oxygen and nutrients (Dejmek et al. 2000).

The finding of higher DNA adduct levels in the infant compared with the mother suggests an increased susceptibility of the developing fetus to DNA damage (Perera et al. 1999). With respect to IUGR, it appears that the increased risk is principally due to exposure to carcinogenic PAHs. This finding is consistent with the idea of a primary role for carcinogenic PAHs in fetal growth modulation (Guyda 1991; MacKenzie and Angevine 1981; Rigdon and Rennels 1964; Zhang et al. 1995). Perera et al. (2003) labeled PAHs as significant independent determinants of birth outcomes. In addition, there appears to be an interaction between exposure to PAHs and genotypes that produce DNA adducts (Whyatt et al. 2001).

Although the specific steps of these pathways need to be further clarified, the molecular epidemiology studies and the similarity of effects of air pollution to those of smoking (Adriaanse et al. 1996; Windham et al. 1999) support the biologic plausibility of the effects.

## Conclusions

Overall, there is evidence implicating air pollution in adverse effects on different birth outcomes, but the strength of the evidence differs between outcomes. The evidence is sufficient to infer a causal relationship between particulate air pollution and respiratory deaths in the postneonatal period. For air pollution and birth weight, the evidence is suggestive of causality, although further studies are needed. For preterm births and IUGR, the evidence as yet is insufficient to infer causality, but the available evidence justifies further studies. Molecular epidemiologic studies suggest possible biologic mechanisms for the effect on birth weight, premature birth, and IUGR and support the view that the relation between pollution and these birth outcomes is genuine. For birth defects, the evidence base so far is insufficient to draw conclusions. In terms of exposure to specific pollutants, particulates seem the most important for infant deaths, and the effect on IUGR seems linked to PAHs, but the existing evidence does not allow precise identification of the different pollutants and the timing of exposure that can result in adverse pregnancy outcomes.

On the basis of this review, we suggest several priorities for future research. First, it remains to be confirmed that the effects on birth weight, prematurity, and IUGR are genuine and causal. Second, it is important to identify the most vulnerable period of exposure in pregnancy. Third, the contribution of different pollutants needs to be established. Fourth, the biologic pathways require further clarification. And finally, with the increasing attention to the life course, it would be interesting to examine whether early exposures and impaired reproductive outcome have any long-term consequences in later life.

## Figures and Tables

**Table 1:** Air pollution and child mortality.

Mortality	Pollutant	Results	Reference
Postneonatal respiratory mortality	TSP	AOR = 2.41 (95% CI, 1.10–5.28) comparing highest vs. lowest quintile	Bobak and Leon 1992
		AOR = 3.91 (95% CI, 0.90–3.50) for 50 μg/m^3^ increase	
Postneonatal respiratory mortality	TSP	AOR = 1.95 (95% CI, 1.90–3.50) for 50 μg/m^3^ increase	Bobak and Leon 1999a
	SO_2_	AOR = 1.74 (95% CI, 1.01–2.98) for 50 μg/m^3^ increase	
	NO_x_	AOR = 1.66 (95% CI, 0.98–2.81) for 50 μg/m^3^ increase	
Postneonatal infant mortality	PM_10_	AOR = 1.10 (95% CI, 1.04–1.16) comparing high vs. low exposure	Woodruff et al. 1997
Respiratory death groups	PM_10_	AOR = 1.40 (95% CI, 1.05–1.85) comparing high vs. low exposure with normal birth weight	
Sudden infant death	PM_10_	AOR = 1.26 (95% CI, 1.14–1.39) comparing high vs. low exposure groups	
Intrauterine mortality	NO_2_	Strong association (coefficient = 0.0013 μg/m^3^, *p* < 0.01)	Pereira et al. 1998
	SO_2_	NE	
	CO	NE	
		Significant association using pollution index NO_x_ + SO_2_ + CO	
	O_3_	NE	
	PM_10_	NE	
Infant mortality	NO_2_	NE	Loomis et al. 1999
	SO_2_	NE	
	CO	NE	
	O_3_	NE	
	PM_10_	6.9% excess (95% CI, 2.5–11.3%) for 10 μg/m^3^ increase	
Perinatal and infant mortality		NE between residence near coke works	Dolk et al. 2000

Abbreviations: AOR, adjusted odds ratio; NE, no effect; PM_10_, particulate matter < 10 μm; TSP, total suspended particulate.

**Table 2:** Air pollution and birth weight.

Outcome	Pollutant	Results	Reference
LBW	SO_2_	AOR = 1.21 (95% CI, 1.06–1.16) for 100 μg/m^3^ increase	Wang et al. 1997
	TSP	AOR = 1.10 (95% CI, 1.05–1.14) for 100 μg/m^3^ increase	
LBW	TSP	OR = 1.04 (95% CI, 0.96–1.12) for 50 μg/m^3^ increase	Bobak and Leon 1999b
	SO_2_	OR = 1.10 (95% CI, 1.02–1.17) for 50 μg/m^3^ increase	
	NO_x_	OR = 1.07 (95% CI, 0.98–1.16) for 50 μg/m^3^ increase	
LBW	NO_x_	NE	Bobak 2000
	SO_2_	AOR = 1.20 (95% CI, 1.11–1.30) for 50 μg/m^3^ increase in the first trimester	
	TSP	AOR = 1.15 (95% CI, 1.07–1.24) for 50 μg/m^3^ increase in the first trimester	
LBW	O_3_	NE	Ritz and Yu 1999
	NO_2_	NE	
	PM_10_	NE	
	CO	OR = 1.22 (95% CI, 1.03–1.44) for CO > 5.5 ppm in the first trimester	
VLBW	TSP + SO_2_	AOR = 2.88 (95% CI, 1.16–7.13) comparing highest vs. lowest exposure groups (56.7 vs .9.9 μg/m^3^)	Rogers et al. 2000
LBW	CO	AOR = 1.43 (95% CI, 1.18–1.74) for 1 ppm increase in first trimester	Maisonet et al. 2001
		AOR = 1.75 (95% CI, 1.50–2.04) for 1 ppm increase in first trimester in African Americans	
	SO_2_	AOR = 1.18/1.20 (95% CI, 1.02–1.36) ppm increase in all trimesters in whites	
LBW	SO_2_ + NO_2_ + PM_10_	AOR = 1.77 (95% CI, 1.00–3.12) comparing petrochemical and control municipalities	Lin et al. 2001b
LBW	CO	AOR = 1.08 (95% CI, 1.04–1.12) in the first trimester	Ha et al. 2001
	NO_2_	AOR = 1.07 (95% CI, 1.03–1.11) in the first trimester	
	SO_2_	AOR = 1.06 (95% CI, 1.02–1.10) in the first trimester	
	TSP	AOR = 1.04 (95% CI, 1.00–1.08) in the first trimester	
LBW	POM	OR = 1.31 (95% CI, 1.21–1.43) comparing highest vs. lowest exposure groups	Vassilev et al. 2001b

Abbreviations: AOR, adjusted odds ratio; NE, no effect; VLBW, very low birth weight (< 1,500 g).

**Table 3:** Air pollution and premature births.

Pollutant	Results	Reference
SO_2_	AOR = 1.21 (95% CI, 1.01–1.45) for 100 μg/m^3^ increase	Xu et al. 1995
TSP	AOR = 1.10 (95% CI, 1.01–1.20) for 100 μg/m^3^ increase	
SO_2_	AOR = 1.27 (95% CI, 1.16–1.39) for 50 μg/m^3^ increase in the 1st trimester	Bobak 2000
TSP	AOR = 1.18 (95% CI, 1.05–1.31) for 50 μg/m^3^ increase in the 1st trimester	
CO	NE	Ritz et al. 2000
NO_2_	NE	
O_3_	NE	
PM_10_	RR = 1.16 (95% 1.06–1.26) for 50 μg/m^3^ increase in the 1st trimester	
SO_2_ + NO_2_ + PM_10_	AOR = 1.41 (91% CI, 1.08–1.82) comparing petrochemical and control municipalities	Lin et al. 2001a

Abbreviations: AOR, adjusted odds ratio; NE, no effect.

**Table 4:** Air pollution and IUGR.

Outcome	Pollutant	Results	Reference
IUGR	SO_2_	NE	Dejmek et al. 1996
	NO_x_	NE	
	O_3_	NE	
IUGR	PM_10_	AOR = 2.64 (95% CI, 1.48–4.71) comparing PM_10_ > 50 μg/m^3^ with PM_10_ < 40 μg/m^3^ in the first month of pregnancy	Dejmek et al. 1999
	PM_2.5_	AOR = 2.11 (95% CI, 1.20–3.70) comparing PM_2.5_ > 37 μg/m^3^ with PM_2.5_ < 27 μg/m^3^ in the first month of pregnancy	
IUGR	PM_10_	AOR = 2.14 (95% CI, 1.42–3.23) comparing PM_10_ > 50 μg/m^3^ with PM_10_ < 40 μg/m^3^ in the first month of pregnancy	Dejmek et al. 2000
	PM_2.5_	AOR = 1.96 (95% CI, 1.02–3.11) comparing PM_2.5_ > 37 μg/m^3^ with PM_2.5_ < 27 μg/m^3^ in the first month of pregnancy	
	carc-PAHs	AOR = 2.15 (95% CI, 1.27–3.63) comparing carc-PAHs > 30 μg/m^3^ with carc-PAHs < 15 μg/m^3^ in the first month of pregnancy	
SGA	POM	AOR = 1.22 (95% CI, 1.16–1.27) comparing highest vs. lowest exposure groups for the term birth	Vassilev et al. 2001a

Abbreviations: AOR, adjusted odds ratio; carc-PAHs, carcinogenic-PAHs; NE, no effect; SGA, small for gestational age.
